# Deep Brain Stimulation for Parkinson’s Disease During the COVID-19 Pandemic: Patient Perspective

**DOI:** 10.3389/fnhum.2021.628105

**Published:** 2021-04-01

**Authors:** Chencheng Zhang, Jing Zhang, Xian Qiu, Yingying Zhang, Zhengyu Lin, Peng Huang, Yixin Pan, Eric A. Storch, Bomin Sun, Dianyou Li

**Affiliations:** ^1^Department of Neurosurgery, Ruijin Hospital, School of Medicine, Shanghai Jiao Tong University, Shanghai, China; ^2^Center for Functional Neurosurgery, Ruijin Hospital, School of Medicine, Shanghai Jiao Tong University, Shanghai, China; ^3^Shanghai Research Center for Brain Science and Brain-Inspired Intelligence, Shanghai, China; ^4^Menninger Department of Psychiatry and Behavioral Sciences, Baylor College of Medicine, Houston, TX, United States

**Keywords:** deep brain stimulation, Parkinson’s disease, COVID-19, Person-centered care, elective surgery

## Abstract

**Background:**

Public health guidelines have recommended that elective medical procedures, including deep brain stimulation (DBS) surgery for Parkinson’s disease (PD), should not be scheduled during the coronavirus (COVID-19) pandemic to prevent further virus spread and overload on health care systems. However, delaying DBS surgery for PD may not be in the best interest of individual patients and is not called for in regions where virus spread is under control and inpatient facilities are not overloaded.

**Methods:**

We administered a newly developed phone questionnaire to 20 consecutive patients with PD who received DBS surgery in Ruijin Hospital in Shanghai during the COVID-19 pandemic. The questionnaire was designed to gather the patients’ experiences and perceptions on the impact of COVID-19 on their everyday activities and access to medical care.

**Results:**

Most of the patients felt confident about the preventive measures taken by the government and hospitals, and they have changed their daily living activities accordingly. Moreover, a large majority of patients felt confident obtaining access to regular and COVID-19-related health care services if needed. Routine clinical referral, sense of security in the hospital during the outbreak, and poor control of PD symptoms were the three main reasons given by patients for seeking DBS surgery during the COVID-19 pandemic.

**Conclusion:**

The COVID-19 pandemic has considerably impacted medical care and patients’ lives but elective procedures, such as DBS surgery for PD, do not need to be rescheduled when the health care system is not overloaded and adequate public health regulations are in place.

## Introduction

The ongoing coronavirus (COVID-19) pandemic is rapidly changing how we live and practice medicine globally. Most public health guidelines developed to deal with the COVID-19 pandemic recommend that inpatient facilities reschedule elective clinical examinations and surgeries as a preventive measure for the virus (Collaborative Covids, 2020b). In line with this general recommendation, many medical centers are postponing elective procedures and deferring non-urgent clinic visits to conserve hospital resources and prevent further spread of COVID-19 (Collaborative Covids, 2020a). The surgical implantation of deep brain stimulation (DBS) electrodes for select patients who suffer from Parkinson’s disease (PD) is considered an elective procedure and hence, should not be scheduled while public health preventive measures for COVID-19 are in place (Gross et al., 2020; Miocinovic et al., 2020). Only patients with PD who have already undergone DBS surgery and encounter a sudden interruption of the implantable pulse generator or a DBS system-related infection are generally viewed as the ones who require urgent hospital care or, in rare cases, emergency surgery (Miocinovic et al., 2020). Yet, rescheduling and delaying DBS surgery for patients who suffer from advanced and medication-refractory PD may not be in the best interest of individual patients.

Public health guidelines for COVID-19 vary across countries, states/provinces, and local municipalities and can change rapidly according to new scientific insights, public health policies, and changing circumstances (del Rio and Malani, 2020). Indeed, public health guidelines initially put forward should not be seen as fixed and universal but need to be continuously updated and adapted to the current situation. Correspondingly, depending on federal and local regulations, the virus spread, and availability of medical resources, some elective and time-sensitive therapeutic procedures may be prioritized in certain regions and hospitals to maintain or reinstate the delivery of regular health care (Thomas et al., 2020), and to admit new patients with PD referred for specialized DBS surgery and treatment. In our hospital, this option was considered because we were not overcrowded with COVID-19 patients and were able to maintain regular health care delivery. Thus, all functional neurosurgeries, as well as face-to-face and remote programming, remained available upon request during the COVID-19 pandemic (Zhang et al., 2020a). Although we were uncertain about the volume of new patients with PD who would be seeking DBS surgery and treatment amidst the COVID-19 pandemic and the implemented public health preventive measures, a substantial number of patients did seek and receive this neurosurgical intervention for PD in our hospital.

We were intrigued by the patients’ perspectives on the COVID-19 pandemic and their own medical risk, as well as on the impact that the COVID-19 crisis may have had on their daily living activities, access to clinical care, and health care costs while they were seeking DBS surgery and treatment for PD during the virus outbreak. Given the uncertain nature of COVID-19 (e.g., future outbreaks) and that other parts of the world not having COVID-19 under control, understanding what PD patients who are seeking DBS think about COVID-19 has important implications for supporting the clinical needs of this sensitive population pre- and post-operatively. In this study, therefore, we examined the perceptions and experiences of a series of PD patients who sought and received DBS surgery and postoperative management in our hospital during the COVID-19 pandemic.

## Methods

### Participants

This study enrolled 20 consecutive patients who had received DBS surgery for PD from February 3, 2020 to April 7, 2020 at the Center for Functional Neurosurgery of Ruijin Hospital in Shanghai. The Ruijin Hospital Institutional Ethical Review Board approved study procedures. All patients had provided written informed consent for the surgical procedure and postoperative follow-ups. Patients admitted to the hospital during the COVID-19 pandemic were mainly local residents because national travel restrictions made it difficult for out-of-town patients to enter the city. They were often accompanied by young family members who, according to national public health policy, could take sufficient vacation to accompany their elderly family members to the hospital for clinical examination and surgical treatment if needed. Hospital appointments had been scheduled during the COVID-19 outbreak and none of the patients opted for rescheduling the surgery.

Initially, a trained health professional called by phone all patients with PD who had undergone DBS surgery during the period of interest and invited them to participate in this survey study. Once a patient accepted the invitation and provided verbal informed consent in the phone call, a structured phone questionnaire was administered by the health professional to acquire information about the patient’s perspective on the COVID-19 pandemic and its impact while he or she was seeking and receiving DBS surgery and treatment for PD. The questionnaire typically took less than 25 min to complete. The phone interviews were done from April 28, 2020 to May 14, 2020.

### COVID-19 Exposure and Impacts Questionnaire

We developed a structured phone questionnaire, referred to as the COVID-19 Exposure and Impacts Questionnaire (CEIQ), to collect information about patient perceptions, attitudes, and experiences in relation to the COVID-19 pandemic (see Tables 1–3). We employed the following four sections of the CEIQ: (1) COVID-19 Personal Status, involving dichotomously scored items about the patients’ health status and medical risk related to the COVID-19 virus; (2) COVID-19 Impact on Living Conditions, consisting of dichotomously or polytomously scored items about the impact of the COVID-19 crisis and associated public health preventive measures on patients’ daily living activities (e.g., occupational or educational functioning), including regular medical visits, and the behavioral changes they personally made to prevent virus infection in public places and at work or home; (3) COVID-19-related Health Care Costs, consisting of dichotomously and quantitatively scored items about patients’ actual or expected direct and indirect personal costs for receiving COVID-19-related health care; and (4) COVID-19 Attitudes and Information, composed of items rated using a 5-point Likert scale (ranging from 1 = strongly disagree to 5 = strongly agree), focusing on patients’ attitudes and perceptions on: (a) the preparedness of the patient self, the city, and the global community for the COVID-19 pandemic; (b) the level of confidence in the city’s preventive approach; (c) the level of trust in COVID-19-related information provided by official sources; (d) the health risk conferred by contracting the virus; and (e) the ability to get access to regular or specialized health care services. Furthermore, we gathered demographic data as well as the main reasons for seeking DBS surgery and treatment during the COVID-19 virus outbreak.

### Hospital Preventive Measures

At the hospital, all patients and accompanying family members were first screened for COVID-19 virus infection. In line with national public health policy, they all possessed carry-on digital codes that documented whether they had traveled to a high-risk area in the past two weeks. All patients also underwent preoperative chest CT screening to detect asymptomatic infections and to ensure that no individuals with COVID-19 were admitted into the general ward. Importantly, management measures (temperature measurement, real-name system recording, and health checks of accompanying personnel) were carried out in single rooms.

Medical personnel’s protection in the hospital was also crucial. We took several measures for enhancing their safety, including the development of nursing guidelines related to COVID-19, along with training, facilitating communication skills, and updating of knowledge on diagnostics, therapeutics, and levels of protection needed to interact with a particular patient. Specifically, first-level protection (wearing disposable surgical masks and disposable head covering) was used for patients with no fever in the ward. Second-level protection measures were used for patients with fever, who received timely referrals to a specialized COVID-19 clinic according to protocol. Also, for some invasive and aerosol-generating procedures, the doctor wore goggles and used closed suction tubes to reduce infection risk. Other preventive measures implemented included standardized procedures for disinfection and physical distancing in operating rooms and rest areas, as well as dividing meals into multiple time slots to reduce the risk of cross-infection.

## Results

### Patient Sample Characteristics

Participants consisted of 20 patients with PD (11 women, 9 men; mean age = 61.4 years, SD = 9.6) (Table 1). Most patients (*n* = 14, 70%) had completed middle school as the highest level of education attained. Also, most patients were married (*n* = 16, 80%) and had either one child (*n* = 10, 50%) or two or three children (*n* = 8, 40%). The majority of patients were retired (*n* = 16, 80%), one patient was employed, and the remaining were unemployed before the COVID-19 pandemic (*n* = 3, 15%). Five patients (25%) reported having experienced mental health problems, mainly anxiety or mood disorder, during their lifetime (Table 1). One patient was treated with medication for anxiety and depression. None of the patients with PD contracted the COVID-19 virus during the hospitalization.

**TABLE 1:** Patients’ demographics and clinical information (*N* = 20).

**Characteristics**	**Value (Percentage)**
**Age (years)**	
mean ± SD	61.4 ± 9.6
range	35–76
Gender (Male/Female)	9/11 (45%/55%)
**Level of education**	
Middle school	14 (70%)
High school/Special secondary school	5 (25%)
Undergraduate	1 (5%)
**Clinical features**	
MDS USPRS-III at med-OFF state	55.8 ± 12.4
MDS USPRS-III at med-ON state	29.3 ± 10.3
BDI-II	14.2 ± 8.6
BAI	11.3 ± 7.9
LEDD (mg)	881.5 ± 406.6
C**ombined household income per year (10 thousand CNY)**	
2–5	1 (5%)
5–10	3 (15%)
10–30	10 (50%)
30–50	3 (15%)
50–100	3 (15%)
**Marital status**	
Single	1 (5%)
Married	16 (80%)
Divorced	1 (5%)
Other	2 (10%)
**Employment status**	
Full-time work	1 (5%)
Retired	16 (80%)
Unable to work	3 (15%)
**Number of children**	
0	2 (10%)
1	10 (50%)
2	6 (30%)
3	2 (10%)
**History of mental disorders**	
No	15 (75%)
Yes	5 (25%)
Generalized anxiety disorder	1
Social anxiety disorder	1
Other anxiety disorder	3
Obsessive-compulsive disorder	1
Depression	2
Bipolar disorder	1
Eating disorder	1

### COVID-19 Exposure and Impacts Questionnaire (Sections 1–3)

Table 2 presents the patients’ CEIQ data involving Section 1 (COVID-19 Personal Status), Section 2 (COVID-19 Impact on Living Conditions), and Section 3 (COVID-19-Related Health Care Costs). Before DBS surgery and at study entry, none of the patients had been infected by the COVID-19 virus or had any other emerging serious infectious disease. One patient, however, received immunosuppressive therapy for diabetes, making this patient at high risk for developing serious or life-threatening COVID-19 symptoms or complications if infected. Seven other patients and one caregiver were similarly at high risk due to advanced age (>65 years) or the presence of a comorbid medical condition.

**TABLE 2:** COVID-19 Exposure and Impacts Questionnaire – Sections 1–3.

**COVID-19 Exposure and Impacts Questionnaire**	**Number (Percentage) for positive response**
**Section 1. COVID-19 personal status**	
1. Have you ever contracted COVID-19?	0 (0%)
2. Do you receive immunosuppressive therapy for respiratory diseases, diabetes or other diseases except for PD?	1 (5%)
3. Is your caregiver a member of an at-risk group for more serious COVID-19 illness (such as being immunosuppressed, over 65 years of age, have pre-existing respiratory disease, diabetes, or other)	1 (5%)
4. Have you previously been impacted by SARS, MERS, H1N1, Ebola, or other serious emerging infectious diseases (that is; you got sick, knew someone who got sick, or lived in an area with cases of the disease)?	0 (0%)
**Section 2. COVID-19 Impact on Living Conditions**	
1. Has your employment been affected by COVID-19?	
Yes, unemployed due to COVID-19 pandemic	0 (0%)
Yes, working hours reduced	0 (0%)
Yes, working hours increased	0 (0%)
Yes, with salary reduction	0 (0%)
Yes, with remote working	0 (0%)
Yes, major events canceled in company or organization	1 (5%)
No, without impact	0 (0%)
Not relevant (retired or unemployed before COVID-19 pandemic)	19% (95%)
2. Have your daily activities been impacted by any of the following?	
Primary/Middle School closures	1 (5%)
University closures	0 (0%)
Transition to online learning	0 (0%)
Inability of being hospitalized or operated in hospital	3 (15%)
Doctor’s appointment canceled or postponed	2 (10%)
Shortage of food and other supplies	5 (25%)
Avoid going to restaurants or stores	8 (40%)
Avoid participating large gatherings (e.g., sport events, cinema)	9 (45%)
Avoid meeting people suspected of having recently visited high-risk areas	6 (30%)
Avoid having international air travel	5 (25%)
Avoid having domestic air travel	5 (25%)
3. Have you voluntarily changed your behaviors due to COVID-19 pandemic?	
Increase the frequency of handwashing	19 (95%)
Use additional or stronger disinfectants/cleaners at home or work	8 (40%)
Consult regularly the websites with COVID-19 information	8 (40%)
Take the disinfectants with you to clean objects that may be contaminated by the virus	4 (20%)
Talk with doctors about health issues related to COVID-19	3 (15%)
Purchase face masks	16 (80%)
Wear the protective mask or other equipment in public	10 (50%)
**Section 3. COVID-19-related Health Care Costs**	
1. Have you incurred any direct costs due to COVID-19 testing and/or treatment?	15 (75%)
If yes, please estimate your direct costs [Median (Range)]	150 (120–300,000) CNY
2. Have you incurred any indirect costs due to COVID-19, e.g., loss of income, additional childcare expenses, costs of necessary travel, preparing for quarantine/isolation?	4 (20%)
If yes, please estimate your indirect costs [Median (Range)]	325 (20–2,000) CNY

The COVID-19 outbreak and its preventive measures profoundly affected the patients’ daily behavior in both public places and their home setting (Table 2). A large proportion of patients reported that they avoided public events (85% of all patients), avoided large gatherings (45%), and avoided restaurants or stores (40%). Many patients also decided to increase their frequency of handwashing (95%), to purchase a face mask (80%), to wear a protective mask or other gear in public (50%), to use extra or stronger disinfectants at home or work (40%), and to visit a web site to gather more information about COVID-19 (40%). Additionally, several patients encountered problems with acquiring food and other product supplies (25%) (Table 2).

Relatively few patients canceled doctor appointments (10%) or were unable to get admitted to the hospital for surgery (15%) during the pandemic. Only a few patients (15%) had talked with a doctor about the health issues related to COVID-19 (Table 2).

Fifteen patients (75%) incurred direct expenditures due to COVID-19 testing or treatment (median = 150 CNY, range = 120–300,000 CNY) (Table 2). Four patients (25%) reported to have incurred indirect expenditures, such as loss of income or extra expenses for necessary travel, due to COVID-19 and associated restrictions (median = 325 CNY, range = 20–2,000 CNY).

### COVID-19 Exposure and Impacts Questionnaire (Section 4)

**FIGURE 1:**
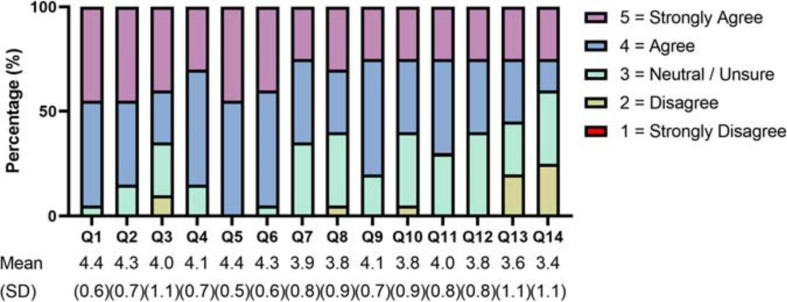
COVID-19 Attitudes and Information. Items were rated using a 5-point Likert scale ranging from 1 (strongly disagree) to 5 (strongly agree). The items are listed below. Q1: I am well prepared for COVID-19. Q2: The local government is well prepared for COVID-19. Q3: The international community is well prepared for COVID-19. Q4: I can access enough information about COVID-19. Q5: I trust information about COVID-19 from official sources. Q6: I am confident that the local government will cope with COVID-19 over the coming months. Q7: The risk of COVID-19 has been exaggerated. Q8: The worst period of the COVID-19 pandemic is over. Q9: I can access the regular (not related to COVID-19) medical care that I need. Q10: If needed, I can easily access COVID-19 testing. Q11: If needed, I can easily access basic medical care for COVID-19. Q12: If needed, I can easily access intensive medical care for COVID-19 (such as hospitalization or respiratory support). Q13: If needed, I can afford treatment for COVID-19. Q14: If needed, I am sufficiently covered by public or private insurance for COVID-19 treatment.

Figure 1 summarizes the patients’ CEIQ data involving Section 4 (COVID-19 Attitudes and Information). Almost all patients (95%) felt that they were well prepared for the COVID-19 outbreak. Most patients also felt that the government (85%) and international community (65%) were well prepared. A large majority of patients (85%) reported to have had access to information about the COVID-19 pandemic (e.g., via websites, television) and all patients (100%) trusted the information provided by official sources. Almost all patients (95%) were confident that the government would adequately handle the virus outbreak within several months, and most patients (60%) believed that the worst of the crisis was over. Surprisingly, most patients (65%) believed that the risk of COVID-19 was exaggerated, but the other patients (35%) were uncertain or neutral about this statement.

Most patients reported that they had easily access to basic COVID-19 medical care (70%) and intensive medical care (60%) if needed, but other patients (30 and 40%, respectively) were not so certain about their access to these special types of health care. Furthermore, about half of the interviewees (55%) reported that they could afford COVID-19 medical care if required, but many patients did not agree (20%) or were unsure (25%). Less than half of the patients (40%) reported that their COVID-19 medical care would be sufficiently covered by the health care system or private health insurance, whereas the other patients were either uncertain (35%) or disagreed (25%) with this statement.

### Reasons for Seeking DBS Surgery

Table 3 presents the main reasons mentioned by the patients for seeking DBS surgery and treatment during the COVID-19 pandemic, along with the personal safety preparations they made before receiving DBS surgery. Clinical referral by the primary health provider, with the hospital appointment scheduled during the pandemic, was the most common reason mentioned among patients (*n* = 10, 50%). Other patients (*n* = 5, 25%) reported that the low regional number of COVID-19 patients and the known well-established protective regimen in the hospital formed the main reason. Four patients (20%) sought DBS treatment because their PD symptoms had become too severe to manage with routine treatment. Finally, most patients (60%) reported that major changes had occurred in their life as a consequence of the COVID-19 pandemic, but the other patients (40%) had experienced no major life changes in relation to the pandemic (Table 3).

**TABLE 3:** Reasons for seeking deep brain stimulation surgery.

**Questions**	**Number (Percentage) for positive response**
1. What’s the main reason for you to choose surgery during the pandemic?	10 (50%)
Doctor appointment with referral	5 (25%)
There are few patients and it is more secure in the hospital	4 (20%)
Poor control of PD symptoms with medical therapy	1 (5%)
Medical insurance referral	1 (5%)
No specific reason	
1. Did you make any special preparations for the surgery, such as taking self-protective measures like wearing a face mask or using antiseptic solution?	7 (35%)

## Discussion

In this study, we examined the perceptions and experiences of a series of elderly patients with PD who sought and received DBS surgery and treatment during the COVID-19 virus outbreak in China. Understanding the perceptions and experiences of this group has important implications for personalizing care and optimizing treatment outcomes. The three main reasons given by the patients for seeking DBS surgery during the virus outbreak were routine clinical referral, personal safety provided by hospital care, and poor control of severe PD symptoms. Most patients felt that they, as well as the government, were well prepared for the COVID-19 virus outbreak. Many patients had changed their behavior accordingly, such as avoiding public events, wearing face masks, and increasing the frequency of handwashing. Moreover, the hospital provided indeed a safe health care setting since none of the patients with PD contracted the COVID-19 virus during hospitalization. Thus, in the context of adequate and timely public health preventive measures, the COVID-19 outbreak did not seem to pose a major obstacle for this series of patients to get access to DBS surgery and treatment for PD. These observations qualify general public health guidelines recommending that elective procedures, such as the surgical implantation of DBS electrodes, should not be scheduled and performed during the COVID-19 pandemic.

Additionally, most patients mentioned that they could get access to regular medical care if needed, despite the COVID-19 outbreak and associated restrictions, but one-fifth of the patients was not sure about this option. Similarly, the majority of patients felt that they had easily access to basic and intensive COVID-19-related medical care if needed, but more than one-third of patients was not so certain about getting access to these special types of health care. Furthermore, a small majority of patients reported that they could afford COVID-19-related medical care if required, but again a substantial portion of patients did not share this view. In relation to the latter observation, such a social- or income-based disparity in health care access should be addressed because lack of access to high-quality health care not only results in poorer patient outcomes but is also a main driver of population-level health disparities and results in higher health care system costs (Wasserman et al., 2019). The patients hold a relatively optimistic view toward the outcome of the COVID-19 outbreak. Almost all patients were confident that the government would adequately control the COVID-19 virus outbreak within a couple of months, and most patients felt that the worst of the crisis was over. The patients had learned of the COVID-19 pandemic from public health officials, along with gathering information via television and internet. Only a few patients had actually talked with a doctor about the health issues related to COVID-19. The latter observation may explain the unexpected finding that most patients felt that the health risk of the COVID-19 virus was exaggerated. This finding is troublesome but illustrates the importance of direct and clear doctor-patient communication addressing the health issues involved and ensuring patients to not underestimate (or overestimate) their own medical risk of contracting the virus (Malecki et al., 2020).

Nevertheless, in contrast to the clinical management and experiences of patients with PD described in this study, the COVID-19 pandemic has profoundly disrupted the delivery of routine clinical care to neurological patients in many other parts of the world. For example, postponed clinical examinations, increased levels of anxiety and depression, and worsening of seizures have been observed in patients with epilepsy during the COVID-19 outbreak and lockdown in Italy (Assenza et al., 2020). The disruption of routine health care provision due to COVID-19 has similarly resulted in clinical worsening and decreased quality of life in many patients with other chronic brain diseases (Lin et al., 2020; Moro and Fernandez, 2020). Fortunately, telemedicine such as virtual visits and remote patient monitoring can help to maintain routine health care during the COVID-19 pandemic by delivering clinical care to neurological patients in their own home setting (Lin et al., 2020; Moro and Fernandez, 2020; Zhang et al., 2020b).

Finally, it should be acknowledged that the present study has certain limitations, which form threats to the external and internal validity of the results. For example, all patients in this study mentioned that they trusted the information about the COVID-19 pandemic provided by official public health agencies. This high level of trust in government is in marked contrast to the lower levels of trust currently evident in many other countries and cultures. Furthermore, in addition to differences in public health policy, the health care system in China differs greatly from the health care systems in other countries. Correspondingly, it remains to be seen to what extent the perspective and experiences of the patients with PD reported in this study can be generalized to other societal cultures and health care systems. Similarly, the study was cross-sectional and included a relatively small number of participants. These study limitations make it uncertain whether the results are robust and can be generalized to patients who live in other areas in China. Moreover, we examined patients with PD who opted for undergoing DBS surgery during the COVID-19 outbreak. This poses another threat to the external validity because these patients who sought and received DBS surgery during the outbreak may have had sufficient financial capacity to control their circumstances and risk of infection. If true, the results cannot be generalized automatically to patients who do not have such financial capacity. Yet, despite its limitations, the present study shows that it is feasible to maintain the delivery of routine pre- and postoperative clinical care to patients with advanced PD who underwent DBS surgery during the COVID-19 pandemic.

## Data Availability Statement

The original contributions presented in the study are included in the article/supplementary material, further inquiries can be directed to the corresponding author/s.

## Ethics Statement

The studies involving human participants were reviewed and approved by Ruijin Hospital Ethics Committee School of Medicine, Shanghai Jiao Tong University. The patients/participants provided their written informed consent to participate in this study.

## Author Contributions

BS, CZ, and ES: study concept and design. XQ, YZ, and ES: questionnaire development. JZ, YZ, and ZL: collection, analysis, and interpretation of data. JZ, XQ, YZ, and CZ: drafting of the manuscript. BS, CZ, ZL, PH, YP, ES, and DL: critical revision of the manuscript. All authors contributed to the article and approved the submitted version.

## Conflict of Interest

The authors declare that the research was conducted in the absence of any commercial or financial relationships that could be construed as a potential conflict of interest.
